# Multi-Platform Characterization of Cerebrospinal Fluid and Serum Metabolome of Patients Affected by Relapsing–Remitting and Primary Progressive Multiple Sclerosis

**DOI:** 10.3390/jcm9030863

**Published:** 2020-03-21

**Authors:** Federica Murgia, Lorena Lorefice, Simone Poddighe, Giuseppe Fenu, Maria Antonietta Secci, Maria Giovanna Marrosu, Eleonora Cocco, Luigi Atzori

**Affiliations:** 1 Department of Biomedical Sciences, Clinical Metabolomics Unit, University of Cagliari, 09121 Cagliari, Italy; simopodd@gmail.com (S.P.); latzori@unica.it (L.A.); 2 Multiple Sclerosis Centre, Department of Medical Sciences and Public Health, Binaghi Hospital, University of Cagliari, via Is Guadazzonis 2, 09126 Cagliari, Italy; lorena.lorefice@hotmail.it (L.L.); giusefenu@gmail.com (G.F.); mariaantonietta.secci@atssardegna.it (M.A.S.); gmarrosu@unica.it (M.G.M.);

**Keywords:** multiple sclerosis, metabolomics, nuclear magnetic resonance, mass spectrometry, biomarkers, pathways analysis

## Abstract

Background: Multiple sclerosis (MS) is a chronic immunemediated disease of the central nervous system with a highly variable clinical presentation and disease progression. In this study, we investigate the metabolomics profile of patients affected by relapsing–remitting MS (RRMS)and primary progressive MS (PPMS), in order to find potential biomarkers to distinguish between the two forms. Methods: Cerebrospinal Fluid CSF and blood samples of 34 patients (RRMS *n* = 22, PPMS *n* = 12) were collected. Nuclear magnetic resonance (^1^H-NMR) and mass spectrometry (coupled with a gas chromatography and liquid chromatography) were used as analytical techniques. Subsequently, a multivariate statistical analysis was performed; the resulting significant variables underwent U-Mann–Whitney test and correction for multiple comparisons. Receiver Operating Characteristic ROC curves were built and the pathways analysis was conducted. Results: The analysis of the serum and the CSF of the two classes, allowed the identification of several altered metabolites (lipids, biogenic amines, and amino acids). The pathways analysis indicated the following pathways were affected: Glutathione metabolism, nitrogen metabolism, glutamine–glutamate metabolism, arginine–ornithine metabolism, phenylalanine, tyrosine and tryptophan biosynthesis etc. Conclusion: The analysis allowed the identification of a set of metabolites able to classify RRMS and PPMS patients, each of whom express different patterns of metabolites in the two biofluids.

## 1. Introduction

Multiple sclerosis (MS) is a chronic immunemediated disease of the central nervous system (CNS), characterized by different levels of structural damage to the brain and spinal cord, including focal demyelinating lesions, axonal loss, and gliosis [1].

Notoriously, MS is characterized by a great heterogeneity in clinical course, neuro-radiological features of the lesions and response to therapy. It affects about 2.5 million people worldwide and is the most common cause of neurologic disability in young and middle-aged adults. MS is twice as common in women as in men. The onset of the disease usually occurs between 20 and 40 years of age with a peak at about 30 years [2]. 

Clinical presentation and course of the disease are highly variable, and several disease types can be recognized: Most patients (85%–90%) with MS begin with a course of relapses and remissions (RRMS) that can evolve in secondary progressive disease (SPMS). Approximately 10 to 15% of patients experience a progressive disease course from onset without any relapses or remissions. These patients are classified as primary progressive MS (PPMS) [3]. A more recent classification re-examined the 1996 clinical course descriptions, categorizing MS patients as relapsing or progressive and active or not active based on clinical relapse rate and MRI findings [4]. 

To date, there is no single clinical manifestation or diagnostic test that is sufficient to diagnose MS: The diagnostic criteria of MS are based on clinical symptoms, MRI features of the demyelinating lesions and paraclinical laboratory tests like laboratory ones (positive oligoclonal bands in the cerebrospinal fluid (CSF) or a raised immunoglobulin G) [5]. Moreover, difficulties in the diagnosis of PPMS, both in terms of distinguishing it from other progressive neurological disorders and excluding patients with previous relapse activity, have recently become an important question in the management of MS patients [6,7,8]. The lack of a single predictive or diagnostic test at most stages of MS disease remains a major obstacle in their phenotypic classification as to personalized MS care. From this perspective, it would be highly beneficial to identify circulating molecules that are highly correlated with inflammatory activity or the severity of neurodegeneration, that can be easily measured with a simple biofluid test and could serve as a biomarker of the disease course, phase, and evolution. 

Metabolomics is based on the systematic study of the complete set of small molecules (metabolites) in a biological system [9]. Metabolites represent the final product of the interaction between gene expression and environmental stimuli, allowing recognition of phenotypic signatures and the possibility of new biomarker targets. Nuclear magnetic resonance (NMR) and mass spectrometry (MS) are the most commonly used techniques for measuring the metabolome [10] in CSF [11] and blood samples [12]. 

To date, several studies have investigated the metabolic profile of MS patients compared to the control patients [12,13] but few studies have investigated the metabolic differences in different subtypes of MS such as RR and PP [14,15,16]. Additionally, no experimental study performed the analysis using both CSF and blood samples while combining several analytical techniques.

In this study, we proposed the investigation of the metabolomics profile of a cohort of patients affected by RRMS and PPMS in order to find potential biomarkers to distinguish between the two forms of MS. For this aim, both CSF and blood samples were collected and ^1^H-NMR and MS (GC-MS and LC-MS) were used as analytical techniques.

## 2. Materials and Methods

Thirty-four CFS and blood samples were collected at the Multiple Sclerosis Centre of the Binaghi Hospital, Cagliari, from patients affected by MS (according to 2017 revisions of the McDonald criteria) [17] with RR (22) and PP (12) course. The study included MS patients with relapsing remitting and progressive course, whose CSF and blood samples were obtained at diagnosis time. Therefore, no influence of DMDs drugs on the metabolite profile of MS patients occurred. In addition, all patients exposed to steroid therapy in the previous 30 days were excluded to exclude this possible confounding factor. Mean values for age and disease duration were 36.8 (SD ± 11.1) and 2.1 (SD ± 1.5) years respectively, while mean expanded disability status scale (EDSS) [18] at sampling time was 1.5 (SD ± 1.1). Table 1 shows the demographic and clinical features of MS patients examined in the study. 

Of the 22 patients with relapsing course, 12 had clinical or neuroradiology activity in the last 3 months, while no disease activity was observed in progressive patients. The study was conducted in accordance with the principles of good clinical practice. The institutional ethics committee of the University of Cagliari approved the study (n° 20/2015), and written informed consent was obtained from each participant. Moreover, the study was conducted in accordance with the Declaration of Helsinki. For each patient, one ml of CSF and 10 mL of blood were previously collected through lumbar puncture and venous sampling at the time of diagnosis. The blood samples and CSF samples were centrifuged at 2500 g for 10 min at 4 °C, and divided into different aliquots for the different analytical analysis (^1^H-NMR, GC-MS and LC-MS). All the samples were stored at −80 °C until analysis.

### 2.1. BiocratesAbsoluteIDQ p180 Kit

CSF and serum samples were analyzed by BIOCRATES Life Sciences AG, Innsbruck, Austria as follows. An aliquot of 50 µL of serum and 30 µL of CSF was analyzed using the Absolute IDQ p180 kit Biocrates (BIOCRATES Life Sciences AG, Innsbruck, Austria). Samples were analyzed using all the pre-analytical and analytical procedures documented and reviewed according to the ISO 9001:2008 certified in-house quality management rules and guidelines. For measuring metabolite concentrations, samples were centrifuged, and the supernatant was used for the analysis. Metabolite concentrations of each sample were determined in a single analysis and samples were placed randomly in the plates. Biocrates commercially available KIT plates were used for the quantification of 180 metabolites in the metabolite classes such as amino acids, acylcarnitines, sphingomyelins, phosphatidylcholines, hexoses, and biogenic amines. The list of the metabolites were reported in Table S1. The fully automated assay was based on PITC (phenylisothiocyanate)-derivatization in the presence of internal standards followed by FIA-MS/MS (acylcarnitines, lipids, and hexose) and LC/MS (amino acids, biogenic amines) using an AB SCIEX 4000 QTrap^®^ mass spectrometer (AB SCIEX, Darmstadt, Germany) with electrospray ionization. Isotope-labeled and chemically homologous internal standards were used for quantification, and in total, 56 analytes were fully validated as absolutely quantitative. The amino acids and biogenic amines were analyzed quantitatively by LC−ESI-MS/MS, with the use of external calibration standards in seven different concentrations and isotope-labeled internal standards for most analytes. The acylcarnitines, glycerophospholipids, sphingolipids, and sum of hexoses were analyzed by FIA-ESI-MS/MS, using a one-point internal standard calibration with representative internal standards (nine isotope-labeled acylcarnitines, one isotope-labeled hexose, one non-labeled lyso-PC, two non-labeled PCs, one nonlabeled SM, a total of 14 internal standards). The experimental metabolomics measurement technique is described in detail by patent US 2007/0004044 [19]. Accuracy of the measurements (determined with the accuracy of the calibrators) was in the normal range of the method (deviations from target ≤ 20%) for all analytes. For sample analysis, validated analytical methods were applied. Quality control samples were within the pre-defined tolerances of the method. Biocrates’ in-house MetIDQ^TM^ software was applied for data export and mapping of measurements with chemical and biochemical background information.

### 2.2. NMR and GC-MS Analysis

#### 2.2.1. Sample Preparation

CSF. A total of 800 µL of each CSF sample were used for the analysis; 600 µL of sample were lyophilized overnight for NMR analysis while 200 µL of CSF were lyophilized overnight for GC-MS analysis. For the NMR analysis, the lyophilized samples were resuspended in 630 µL D2O + 70 µL of 1.5 mM phosphate buffer with 5.85 mM trimethylsilylpropanoic acid (TSP), and 650 µL of the solution was transferred in the NMR tube. For the GC-MS analysis, dried extracts were derivatized with 50 μL of methoxyamine dissolved in pyridine (10 mg/mL) (Sigma-Aldrich, St. Louis, MO, USA) at 70 °C. After 1 h 100 μL of N-Methyl-N-(trimethylsilyl)-trifluoroacetamide, (MSTFA, Sigma-Aldrich, St. Louis, MO, USA) were added and left at RT for 1 hr. The samples were then successively diluted in 100 μL of hexane (Sigma-Aldrich, St. Louis, MO, USA).

Serum: The serum samples were extracted as previously described [20]. Briefly, serum samples were thawed and centrifuged at 2500 g for 10 min at 4 °C. An 800 µL aliquot was added to 2400 µL of a chloroform/methanol 1:1 plus 350 µL of distilled water. The samples were vortexed for 1 min and centrifuged for 30 min at 1700× *g* at RT. The hydrophilic and hydrophobic phases were obtained. The water-phase was divided in 2 aliquots, concentrated overnight using a speed vacuum centrifuge for GC-MS and ^1^H-NMR analysis. For the NMR analysis, the concentrated water-phase was resuspended in 630 µL of D_2_O, 70 µL of 5.07 mM trimethylsilylpropanoic acid (TSP). TSP was added to provide an internal reference for the chemical shifts (0 ppm), and 650 µL of the solution were transferred to a 5 mm NMR tube. For the GC-MS analysis derivatization was undertaken by adding 100 μL of methoxyamine hydrochloride in pyridine solution (10 mg/mL) to dried samples for 17 h. Subsequently, 100 μL of N-trimethylsilyltrifluoroacetamide (MSTFA) were added and vortexed atRT, 1 hr. Samples were then diluted in hexane (600 μL) with an internal standard (undecane at 25 ppm). Diluted samples were then filtered (PTFE 0.45 μm) and transferred into glass vials. By following the same procedure, samples blanks were made used for the samples to avoid background noise resulting from the chemicals used for the preparation and the laboratory instruments.

#### 2.2.2. NMR Analysis and Data Processing

The samples were analyzed with a Varian UNITY INOVA 500 spectrometer (Agilent Technologies, Inc., Santa Clara, CA, USA), which was operated at 499 MHz equipped with a 5 mm triple resonance probe with z-axis pulsed field gradients and an auto-sampler with 50 locations. One-dimensional ^1^H-NMR spectra were collected at 300 K with a NOESY pulse sequence for the CSF samples and PreSat sequence for the serum samples to suppress the residual water signal. The spectra were recorded with a spectral width of 6000; a frequency of 2 Hz; an acquisition time of 1.5 s; a relaxation delay of 2 ms; and a 90° pulse of 9.2 µs. The number of scans was 256. Each Free Induction Decay (FID) was zero-filled to 64 k points and multiplied by a 0.5 Hz exponential line-broadening function. The spectra were manually phased and baseline corrected. By using Mestre Nova software (version 8.1, Mestrelab Research S.L. Santiago de Compostela, Spain)) each NMR spectrum was divided into consecutive “bins” of 0.04 ppm. The serum spectral area investigated was the region between 0.6 and 8.6 ppm. The regions between 4.60 and 5.2 ppm and between 5.24 and 6.6 ppm were excluded to remove variations in the pre-saturation of the residual water resonance and spectral regions of noise. The CSF spectral area investigated was the region between 0.64 and 6.4 ppm. To minimize the effects of the different concentrations of serum/CSF samples, the integrated area within each bin was normalized to a constant sum of 100. 

#### 2.2.3. GC-MS Analysis and Data Processing

One microliter of derivatized sample underwent splitless injection into a 7890A gas chromatograph coupled with a 5975C Network mass spectrometer (Agilent Technologies, Santa Clara, CA, USA) equipped with a 30 m × 0.25 mm ID, fused silica capillary column, with a 0.25 μM TG-5MS stationary phase (Thermo Fisher Scientific, Waltham, MA, USA). The injector and transfer line temperatures were at 250 °C and 280 °C, respectively. The gas flow rate through the column was 1 mL/min. The column initial temperature was kept at 60 °C for 3 min, then increased to 140 °C at 7 °C/min, held at 140 °C for 4 min, increased to 300 °C at 5 °C/min and kept for 1 min. Identification of metabolites was performed using the standard NIST 08 [21] (http://www.nist.gov/srd/mslist.cfm), Fiehn 2013 [22] (http://fiehnlab.ucdavis.edu/Metabolite-Library-2007) and GMD [23] (http://gmd.mpimp-golm.mpg.de) mass spectra libraries (match ≥ 40%) and, when available, by comparison with authentic standards. Data processing was performed by using a pipeline in Knime [24]. In brief, peak detection and deconvolution were performed in a R-XCMS package, filtering was performed using blank samples and keeping features present in ≥50% of the samples. Missing value imputation was conducted using a random forest algorithm. Relative concentrations of the discriminant metabolites were obtained by the chromatogram area and then normalized by median fold change.

### 2.3. Statistical Analysis

A multivariate statistical analysis was performed on the matrix resulting by LC-MS/MS, FIA-MS/MS, ^1^H-NMR, and GC-MS using SIMCA-P software (ver. 15.0, Umetrics, Sweden). The variables were Pareto scaled to emphasize all metabolite signals and reduce the spectral noise for the ^1^H-NMR analysis and UV scaled for the MS analysis.

The initial data analyses were conducted using the Principal Component Analysis (PCA), which is important for the exploration of the sample distributions without classification. To identify potential outliers, the DmodX and Hotelling’s T2 tests were applied. 

Partial least square discriminant analyses (PLS-DA) were subsequently applied. PLS-DA maximize the discrimination between samples assigned to different classes. The variance and the predictive ability (R^2^X, R^2^Y, Q^2^) were established to evaluate the suitability of the models. PLS-DA models were performed by using only variables corresponding to VIP (Variable Influence on Projection) value > 1, as a quantitative estimation of the discriminatory power of each individual metabolite. Variables with VIP > 1 are the most relevant for explaining Y (assignment of two classes) [25]. 

In addition, a permutation test (*n* = 400) was performed to validate the models. The scores from each PLS-DA model were subjected to a CV-ANOVA to test for significance (*p* < 0.05).

The most significant variables were extracted by the loading plot from each model and for the ^1^H^-^NMR data were identified using the Chenomx NMR Suite 7.1 (Chenomx Inc., Chenomx Inc., Edmonton, Alberta, Canada) [26]. GraphPad Prism software (version 7.01, GraphPad Software, Inc., San Diego, CA, USA) was used to perform the univariate statistical analysis of the data. To verify the significance of the metabolites resulting from multivariate statistical analysis U-Mann–Whitney test a Holm–Bonferroni test, to correct for multiple comparisons were performed. Subsequently, to test the sensitivity and specificity of these metabolites, Receiver Operating Characteristic ROC curves were built; this is generally considered the standard method for performance assessment of target biomolecules [27]. ROC curves were built using the concentrations of the metabolites with *p*-value < 0.05 as input, with the aim of testing their sensitivity and specificity in classifying the patients by using GraphPad Prism software (version 7.01, GraphPad Software, Inc., San Diego, CA, USA).

### 2.4. Pathways Analysis 

Metabolic pathways were generated by using MetaboAnalyst 4.0 (MetaboAnalyst 4.0, Xia Lab. Ste. Anne de Bellevue, Quebec) a web server designed to obtain a comprehensive metabolomic data analysis, visualization and interpretation [28]. This approach permits to correlate metabolites changes with metabolic networks. In particular, the pathway analysis module of Metaboanalyst 4.0 combines results from powerful pathway enrichment analysis with pathway topology analysis to help researchers identify the most relevant pathways involved in the conditions under study. MetaboAnalyst performs in-house mapping of common compound names to a wide variety of database identifiers including KEGG, HMDB, ChEBI, METLIN, and PubChem prior to performing any functional analysis. In our analysis, metabolites having *p*-value < 0.05 were used. Only pathways having *p*-value < 0.05 were considered for the discussion and biological interpretation.

## 3. Results

The data generated by all the analytical technique used for the analysis of the CSF and serum were organized in matrix that underwent to multivariate statistical analysis. Firstly, a multivariate analysis was performed on the results from NMR and GC-MS. For the NMR analysis, the total number of variables obtained (bins) was 103 for the CSF, and 155 for the serum, while for the GC-MS analysis of the CSF, the total number of variables obtained was 35, and 40 for the serum. The generate models did not show any significant difference between RRMS and PPMS (low Q^2^ and *p* > 0.05, see Figure S1 and Table S2). and for these reasons the VIPs were not taken into consideration. 

**Figure 1:**
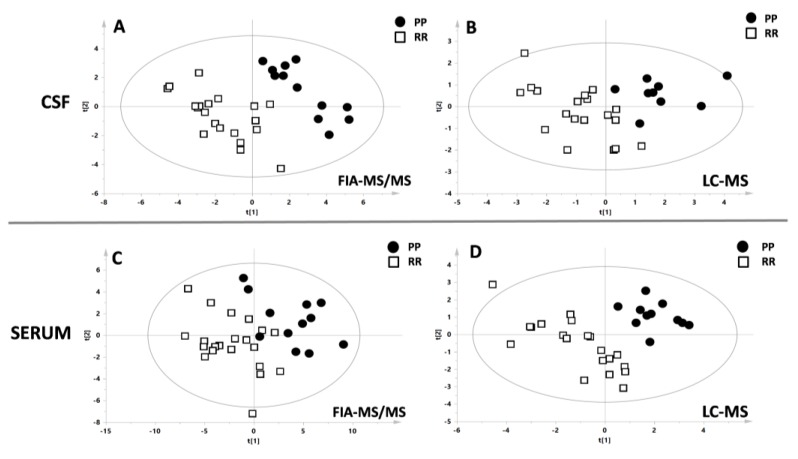
**Multivariate analysis. A**–**B**: PLS-DA models resulted from the analysis of cerebrospinal fluid (CSF) samples with FIA-MS/MS and LC-MS. **C**–**D**: PLS-DA models resulted from the analysis of serum samples with FIA-MS/MS and LC-MS. Black circles indicate primary progressive MS (PPMS) patients while white boxes indicate RRMS patients. The statistical parameters were all significant and were reported in the Table 1.

Subsequently, multivariate analysis of the matrix from FIA-MS/MS (acylcarnitines, lipids, and hexose) and LC/MS (amino acids, biogenic amines) was applied to test the possible differences between RRMS vs PPMS. A separation of the samples, in line with the presence of the different types of MS, was observed by the application of the supervised model PLS-DA (Figure 1) with good statistical parameters. All the parameters of the models were reported in the Table 2. Based on the value of the VIP (>1), 49 metabolites belonging to the classes of acylcarnitines, glycerophospholipids, sphingolipids and 12 metabolites belonging to the classes of the amino acid and biogenic amines were included for the CSF’s PLS-DA models.

Similarly, for the serum, based on the value of the VIP (>1), 50 metabolites belonging to the classes of acylcarnitines, glycerophospholipids, sphingolipids, and 16 metabolites belonging to the classes of amino acids and biogenic amines were included in the PLS-DA models.

**Figure 2:**
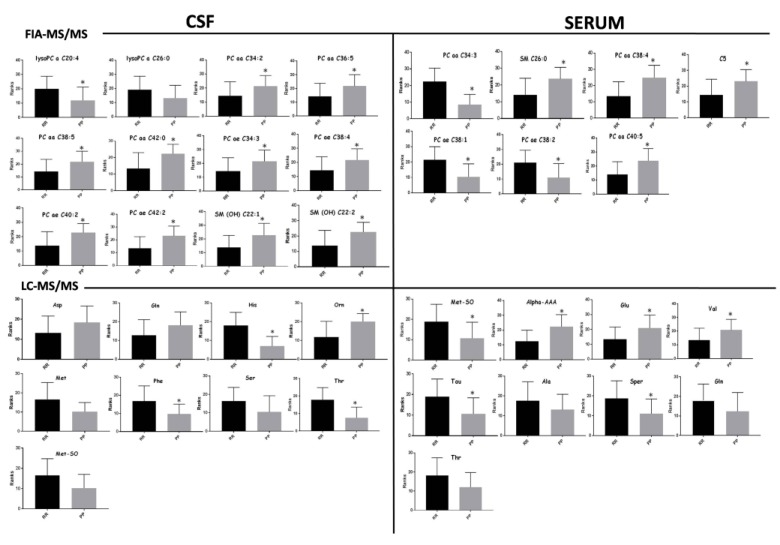
Univariate analysis. Bar-plot of the most discriminant metabolites resulting from the analysis of CSF and serum analyzed with FIA-MS/MS and LC-MS/MS. The black bar indicates the average concentration of the relapses and remissions (RRMS) class expressed as ranks, while the grey bar indicates the average concentration of the PPMS class expressed as ranks. Stars indicate significant change in the concentration of the metabolites.

**Figure 3:**
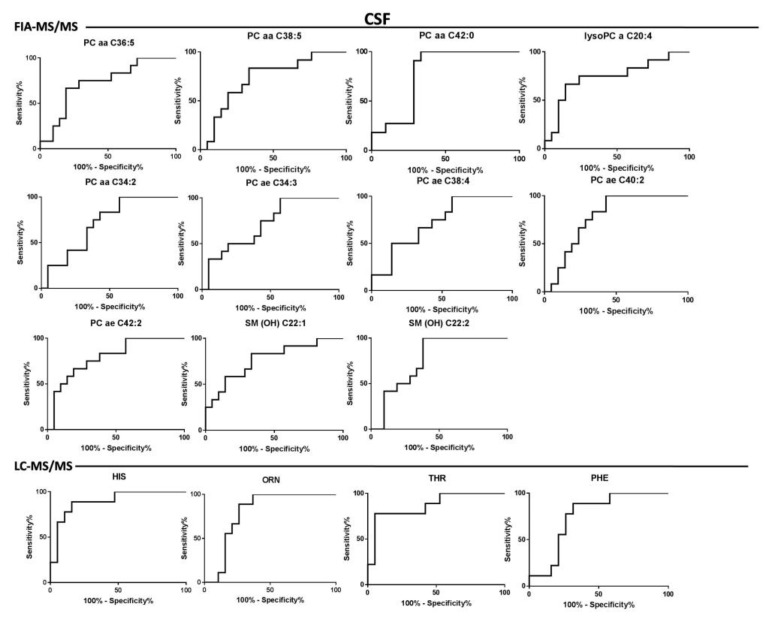
CSF Biomarkers evaluation. Roc curves of the most important metabolites resulting from the multivariate analysis of the CSF matrix, generated with FIA-MS/MS and LC-MS/MS respectively.

**Figure 4:**
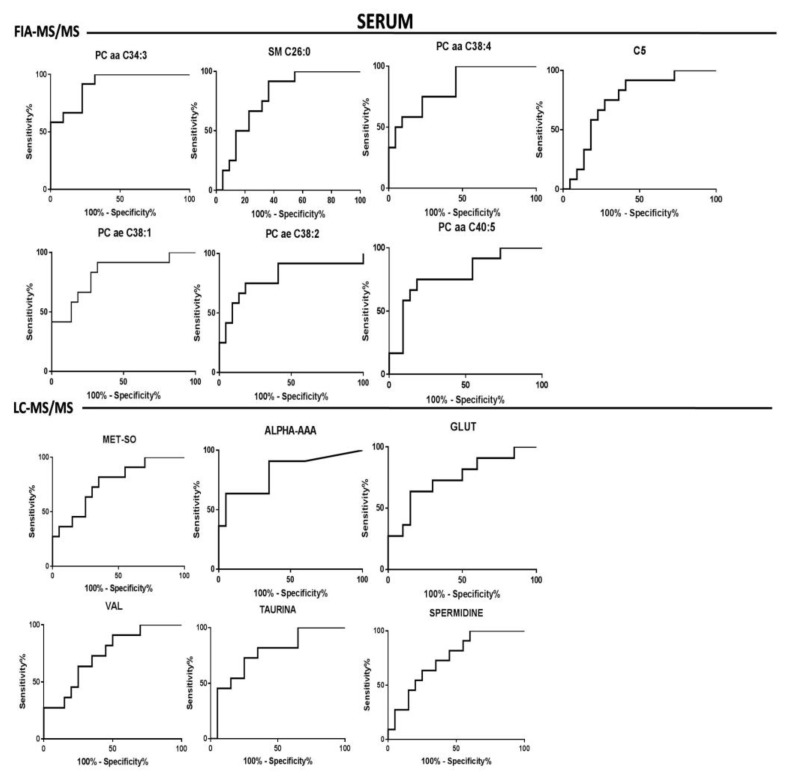
Serum biomarkers evaluation. Roc curves of the most important metabolites resulting from the multivariate analysis of the serum matrix, generated with FIA-MS/MS and LC-MS/MS respectively.

The goodness of fit of the models allowed the possibility of identifying the discriminant variables responsible of RRMS and PPMS by exploiting the information coming from the loadings plot and VIPs values. Box plots of the most important metabolites (having VIPs values > 1) are represented in Figure 2. Moreover, the different concentrations of the discriminant metabolites for each class were tested through the U-Mann–Whitney test and subsequently, for those metabolites having *p*-value < 0.05, a Holm–Bonferroni correction for multiple comparisons was applied. Furthermore, metabolites that exhibited the greatest differences between the studied groups according to a *p*-value < 0.05 were selected to create the ROC curve. The univariate analysis revealed that all the metabolites from the analysis of the serum by LC-MS/MS and FIA-MS/MS resulting significant after U-Mann–Whitney test, passed the Holm–Bonferroni correction (PC aa C34:3, PC aa C38:4, PC ae C38:1, PC ae C38:2, PC aa C40:5, SM C26:0, C5, Methionine-Sulfoxide, alpha-Aminoadipic acid, glutamate, valine, taurine, spermidine). In the case of CSF analysis, 1 phosphocholine (PCae C42:2) from the FIA-MS/MS analysis and histidine, ornithine, phenylalanine and threonine from the LC-MS/MS passed the correction for multiple comparison. These metabolites have been considered suitable as biomarkers for the classification of the two types of MS. In this light, ROC curves were performed to test their sensitivity and sensibility. A synthesis of the significantly altered metabolites resulting from the analysis of the CSF and the serum is reported in Table 3 and in Table 4, respectively. The tables indicate: trends of metabolites in the RR and PP classes (+ or -), *p*-value after U-Mann–Whitney test, *p*-value after Holm–Bonferroni correction and statistical data of the ROC-curve (area under the curve, standard error, confidential interval, *p*-value). ROC curves of the discriminant metabolites in CSF and serum were reported in Figure 3 and Figure 4, respectively.

To avoid the confounding effect, due to the different age of the patients of the two classes, Spearman Correlation was perform relating the selected metabolites and the age of the patients. A weak correlation was found for the ornithine (R^2^ = 0.5), PC ae C42:2 (R^2^ = 0.46) and histidine (R^2^ = −0.44) in the CSF, while for the serum metabolites PC aa C34:4 (R^2^ = −0.48), taurine (R^2^ = −0.55), alpha AAA(R^2^ = 0.41) and spermidine (R^2^ = −0.41) showed a weak correlation. All the results are shown in Figures S2 and S3.

**Figure 5:**
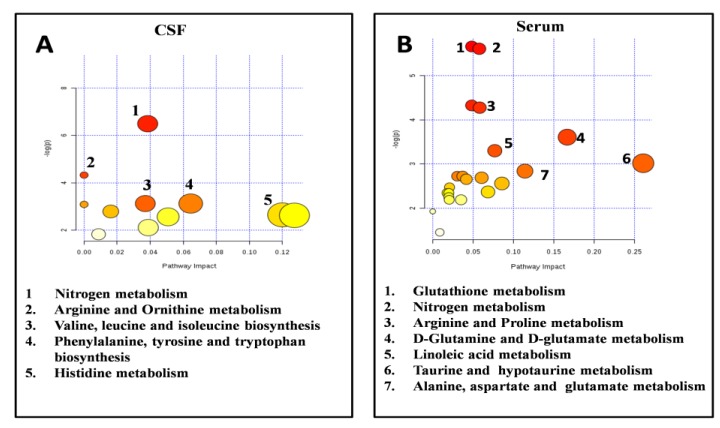
Pathways analysis. Pathways analysis of the most discriminant metabolites passing the Holm–Bonferroni correction in CSF and serum. (**A**) Most altered pathways between RR and PP patients in CSF samples: nitrogen metabolism, arginine and ornithine metabolism, branched chain amino acid (BCAAs) biosynthesis, phenylalanine, tyrosine and tryptophan biosynthesis and histidine metabolism. (**B**) Most altered pathways between RR and PP patients in serum samples: glutathione metabolism, nitrogen metabolism, arginine and proline metabolism, glutamine and glutamate metabolism, linoleic acid metabolism, taurine and hypotaurine metabolism and, finally, alanine, aspartate, and glutamate metabolism.

The metabolites passing the Holm–Bonferroni correction were considered the most relevant for the classification of the RRMS and PPMS classes. The subsequent step was to investigate the biological meaning of the selected metabolites by using the Metaboanalyst tool. The pathway analysis algorithm was based on Fisher’s Exact Test and Out-degree Centrality for the Pathway Topology Analysis. As shown in Figure 5, nitrogen metabolism, arginine and ornithine metabolism, branched chain amino acid (BCAAs) biosynthesis, phenylalanine, tyrosine and tryptophan biosynthesis and histidine metabolism were the most altered pathways between the two classes of patients in CSF. The most altered pathways between RR and PP resulting from the analysis of the serum metabolites were glutathione metabolism, nitrogen metabolism, arginine and proline metabolism, glutamine and glutamate metabolism, linoleic acid metabolism, taurine and hypotaurine metabolism and, finally, alanine, aspartate, and glutamate metabolism. 

The best similarities of the results between CSF and blood were common altered pathways such as pathways linked the oxidative stress (Glutathione metabolism and nitrogen metabolism) and the arginine metabolism, while differences were characterized by changes in different amino acid pathways (glutamine and glutamate metabolism, taurine, and hypotaurine metabolism and alanine, aspartate metabolism in serum; branched chain amino acid, phenylalanine, tyrosine, tryptophan biosynthesis and histidine metabolism in CSF).

## 4. Discussion

During previous decades, the discovery of reliable biomarkers for MS proved very difficult, due to the clinical and pathophysiological complexities of the disease. In this context, the “-Omics” technologies offer the opportunity of large-scale analysis and identification of new candidate biomarkers at multiple levels of cell biology. The goal of the study was to characterize the metabolomics profile of serum and CSF samples of patients affected by different MS sub-types: RR and PP. Several analytical techniques, were used to determine the concentration of a large number of potential biomarkers. Subsequently, different statistical methods were applied in order to identify the discriminant metabolites able to classify patients based on their subtypes of MS. In this study. both ^1^H-NMR and GC-MS did not allow identification a specific metabolic fingerprint of the RR and PP patients in CSF and serum. Thus, we focused our attention on the results from the MS/MS analysis.

Actually, there is no diagnostic laboratory test available that fulfils the criteria of a complete diagnosis in multiple sclerosis, often resulting in delayed definitive diagnosis [29]. The search for biomarkers in this area is very active. but very few molecules have been rigorously validated and used in clinical practice [30,31]. In our study, we chose to investigate the metabolome of both CSF and blood samples. For diagnostic, prognostic, and therapeutic properties, CSF offers a unique opportunity to sample the metabolic content of fluid circulating around the brain and cerebrovascular interfaces reflecting directly brain activity. This may provide important information about neurological damages induced by the progression of MS [31], while blood analysis represents a non-invasive method to investigate peripheral pathological alterations. The analysis of the serum with the FIA-MS/MS and LC-MS/MS allowed the identification of PC aa C34:3 (AUC = 0.91. *p*-Value after Holm–Bonferroni correction 0.001) as the best lipid compound to classify the two groups, while alpha-AAA was the most discriminant between the amino acids and biogenic amines (AUC = 0.81, *p*-Value after Holm–Bonferroni correction 0.01). Moreover, the analysis of the CSF allowed the identification of PC ae C42:2 (AUC = 0.79, *p*-value after Holm–Bonferroni correction 0.04) as the best lipid compound able to classify the patients, while between the amino acids and biogenic amines the most discriminant was histidine (AUC = 0.89, *p*-Value after Holm–Bonferroni correction 0.001). The analysis of the samples with the NMR and GC-MS did not found any significant differences between PPMS and RRMS both in CSF and serum. The serum of a similar cohort of patients (three subtypes of MS, RRMS, SPMS, and PPMS) was analyzed by Dickens et al. [15] with the NMR: They obtained a good results for the comparison between RRMS and SPMS but, as found by our investigation, they underlined the lack of a predictive model between the PP and RR patients.

The discriminant metabolites were studied for a better understanding of the pathophysiology of the two sub-types of MS, by identifying the altered metabolic pathways in RR and PP. Unfortunately, few metabolomics studies based on the different subtypes of MS are present in literature, so is not simple to fully understand our results. Glutathione metabolism, nitrogen metabolism, arginine and proline metabolism, glutamine and glutamate metabolism, linoleic acid metabolism, taurine and hypotaurine metabolism and alanine, aspartate and glutamate metabolism were the most altered in serum between the two classes. In the CSF, nitrogen metabolism, arginine and ornithine metabolism, branched chain amino acid (BCAAs) biosynthesis, phenylalanine, tyrosine and tryptophan biosynthesis and histidine metabolism were the most altered pathways. Stoessel et al. [14] comparing the plasma of PPMS with RRMS and controls patients found the alteration of several pathways such as, linoleic acid metabolism, arginine and proline metabolism, phenylalanine, tyrosine and tryptophan biosynthesis and nitrogen metabolism perfectly in line with our results. Moreover, in the same study, they found a decline in levels of LysoPC (20:0) during the disease course of PPMS, but we did not found any significant differences of this lysoPC comparing the two classes of patients.

Glutathione metabolism and nitrogen metabolism (that we found altered in both CSF and blood) are closely linked to oxidative stress. Oxygen and nitrogen free radicals may represent important features in the pathogenesis of MS. Radical oxygen species (ROS) are particularly active in the brain and neuronal tissue and are bio-products by the metabolism of excitatory amino acids and neurotransmitters. They are generated by constant use of oxygen in the mitochondria to supply the energy, and by many enzymatic and non-enzymatic pathways [32]. ROS attack glia and neurons leading to neuronal damage such as demyelination and axonal injury. In addition, free radicals can activate certain transcription factors (transcription factor-kappa B. NF-κB), promoting the up-regulation of the expression of many genes involved in MS, (tumor necrosis factor-α, nitric oxide synthase, iNOS, intracellular adhesion molecule 1, ICAM-1, etc.) [33]. The fundamental role of oxidative stress in MS has been proved by several scientific studies that found evidence of lipid peroxidation in the CSF and in the plasma of patients with MS [34]. Moreover, it has been proposed that reactive nitrogen species promote myelin and oligodendrocyte destruction due to their cytotoxic effects on nerve and glial cells [35]. Numerous studies had shown increased free radical activity, and/or deficiencies in important antioxidant enzymes in patients with MS compared with healthy controls [36]. From the metabolomics point of view, several study demonstrate the key role of metabolites linked to the presence of oxidative stress in MS [37,38]. Interestedly, Koch et al. analyzed the serum and peripheral blood leukocytes from patients with benign relapsing remitting MS, secondary progressive MS, primary progressive MS and healthy controls found increased ROS formation occurs in all subgroups of MS, but the highest production of ROS was found in patients with PPMS [39].

Other altered pathways in serum were glutamine and glutamate metabolism, as well as alanine, aspartate and glutamate metabolism. These are excitatory amino acids (EAA), fundamental for synaptic connection. Brain excitotoxicity is the result of an imbalance between excitatory processes and GABA/glycine-mediated inhibitory processes, caused by glutamate overload. Glutamate is the major EAA and both intracellular and extracellular physiological concentrations are precisely controlled [40]. Glutamate overload most frequently results from the glutamate–glutamine cycle dysfunction leading to a damage to neurological tissue due to overstimulation of glutamate receptors, and subsequent excitotoxic injury of neurons and glial cells, as widely demonstrated [41,42]. Considering the pivotal role of the glutamate in the CNS, it is not surprising its relevance also in a pathological conditions such as MS [43,44]. Interestingly, Sarchielli et al. demonstrated an increase level of glutamate and aspartate in the CSF of patients with RR MS during relapse and even during a stable clinical phase. Also, in the patients with SPMS, an increase in the CSF levels of glutamate and aspartate emerged compared with those in the control subjects, particularly in patients with a progression of neurological impairment [45].

In addition, in our data, the arginine metabolism pathway was altered in both serum and CSF. As demonstrated by several studies, altered arginine metabolism represent a typical feature in MS [46,47], in both human and in animal models [48]. Indeed, the pathogenesis of MS is based on two major theories: An autoimmune and a neurodegenerative mechanism. The neurodegenerative hypothesis involves metabolic changes in the constituents of myelin, which results in destabilization of membrane architecture and myelin degradation [49]. One of the modifications involves the conversion of peptide bound arginine to peptide bound citrulline, an enzymatic reaction called deimination [50,51]. Moreover, arginine is the precursor to nitric oxide in a reaction catalyzed by the nitric oxide synthase family [46]. Therefore, alteration of arginine metabolism, that we found significant in our analysis, could affect nitric oxide synthesis and be involved in oxidative stress. The role of amino acids in MS is suggested by the involvement of several amino acid systems, as our findings demonstrate. Also, BCAAs participate both directly and indirectly in a variety of important biochemical functions in the brain. These include protein synthesis, production of energy, and compartmentalization of glutamate [52]. Moreover, BCAAs are a known source of pyruvate for energy metabolism, and de novo synthesis of macromolecules within neural and immune cells [53,54].

Phenylalanine, tyrosine, and tryptophan biosynthesis was an altered pathway from the analysis of the CSF. Tryptophan is closely linked to the kynurenine pathway, which is activated in number of inflammatory and neurodegenerative diseases, including MS, and as such represents a common pathological mechanism highly relevant for the understanding of MS pathology [55]. Accumulating evidences demonstrates the involvement of tryptophan metabolism, in particular activation of the kynurenine pathway in neurocognitive disorders under CNS inflammatory conditions such as MS [56,57,58]. Moreover, this net was evaluated also in the comparison between RRMS and the progression disease subtypes SPMS and PPMS, indicating the key role of the metabolites involved in the tryptophan metabolism. This study reveals how the ratio kinurenic acid/quinolic acid was increased in SPMS and PPMS compared to the RRMS, directly linked to an excitotoxicity effect. [16]

Metabolomics represents an innovative approach able advance discover new potential biomarkers and to explore pathophysiological features in a pathological condition. We believe that the use of this new tools will help us come to a greater understanding of the pathogenesis of MS, improving its diagnosis, its classification, availability of effective treatment and to define the response to therapy, as recent investigations have shown [59]. Despite the weak correlation between the age of the patients and the concentration of the selected metabolites, age could be considered as a confounder factor, and its contribution should not be overlooked. Further investigations in larger cohorts are necessary to confirm our preliminary results and to explore the metabolomics fingerprint related to MS and its evolution. Moreover, the evaluation of a control group, that it was not possible to recruit for ethical reasons, would have been useful. This aspect might represent a point of limitation for the study.

## Figures and Tables

**Table 1:** Demographic and clinical features of multiple sclerosis (MS) patients included in the study.

	MS Patients (34)	Relapsing (22)	Progressive (12)	*p*-Value
Male Gender	14 (41.2%)	6 (27.2%)	8 (66.6%)	ns
Age (mean ± SD) years	37.3 ± 12.8	32±8.4	47.1 ± 12.8	<0.05
MS Disease Duration (mean ± SD) years	2.1 ± 1.5	1.2 ± 1.4	3.7 ± 1.2	<0.05
Expanded Disability Status Scale (EDSS) score	2.1 ± 1.1	1.1 ± 1.5	3.9 ± 1.7	<0.05

**Table 2:** Statistical parameters of the multivariate models resulting from the analysis of the matrix generated by FIA-MS/MS and LC-MS analysis.

	CSF					SERUM				
	R^2^X	R^2^Y	Q^2^	*p*-Value	Permutation Test: Intercept R^2^\Q^2^	R^2^X	R^2^Y	Q^2^	*p*-Value	Permutation Test: Intercept R^2^\Q^2^
**FIA-MS/MS**	0.272	0.862	0.634	2,6e-05	0.59/−0.23	0.523	0.666	0.512	0.0004	0.33/−0.19
**LC-MS**	0.395	0.697	0.496	0.002	0.29/−0.28	0.224	0.846	0.514	0.0002	0.35/−0.26

**Table 3:** Trend, univariate analysis, and ROC curve analysis of the discriminant metabolites in CSF samples.

	CSF							
	METABOLITES	RR vs. PP	*p*-Value	Holm–Bonf. Correction	ROC-CURVE			
					AUC	Std. Error	CI	*p*-Value
FIA-MS/MS	-lysoPC a C20:4	+	0.02	ns	0.74	0.09	0.55–0.93	0.02
	-PC aa C34:2	-	0.04	ns	0.71	0.08	0.53–0.88	0.04
	-PC aa C36:5	-	0.03	ns	0.72	0.09	0.54–0.90	0.03
	-PC aa C38:5	-	0.02	ns	0.73	0.09	0.55–0.90	0.03
	-PC aa C42:0	-	0.009	ns	0.78	0.08	0.61–0.94	0.01
	-PC ae C34:3	-	0.04	ns	0.71	0.09	0.53–0.89	0.04
	-PC ae C38:4	-	0.03	ns	0.72	0.08	0.55–0.89	0.03
	-PC ae C40:2	-	0.007	ns	0.78	0.08	0.62–0.93	0.008
	PC ae C42:2	-	0.004	0.04	0.79	0.07	0.64–0.95	0.005
	SM(OH) C 22:1	-	0.010	ns	0.77	0.08	0.6–0.93	0.01
	SM(OH) C 22:2	-	0.01	ns	0.76	0.08	0.6–0.92	0.01
LC-MS/MS	HIS	+	0.0004	0.001	0.89	0.06	0.77–1	0.0009
	ORN	-	0.01	0.010	0.79	0.08	0.63–0.96	0.03
	PHE	+	0.03	0.010	0.75	0.09	0.57–0.93	0.03
	THR	+	0.001	0.002	0.86	0.07	0.71–1	0.002

+ or - indicates a higher or lower level of the metabolite in RR vs PP group.

**Table 4:** Trend, univariate analysis, and ROC curve analysis of the discriminant metabolites in serum samples

	SERUM							
	METABOLITES	RR vs. PP	*p*-Value	Holm––Bonf. Correction	ROC-CURVE			
					AUC	Std. Error	CI	*p*-Value
**FIA-MS/MS**	PC aa C34:3	+	<0.0001	0.001	0.91	0.05	0.81–1.00	<0.0001
	PC aa C38:4	-	0.0010	0.005	0.83	0.07	0.70–0.97	0.001
	PC ae C38:1	+	0.0016	0.006	0.82	0.08	0.67–0.97	0.002
	PC ae C38:2	+	0.0036	0.011	0.80	0.08	0.62–0.97	0.004
	PC aa C40:5	-	0.0059	0.012	0.78	0.08	0.61–0.95	0.007
	SM C26:0	-	0.006	0.012	0.79	0.08	0.63–0.93	0.008
	C5	-	0.0149	0.012	0.75	0.08	0.6–0.92	0.015
**LC-MS/MS**	MET-SO	+	0.010	0.040	0.76	0.08	0.59–0.94	0.01
	ALPHA-AAA	-	0.002	0.010	0.81	0.08	0.65–0.98	0.003
	GLU	-	0.02	0.040	0.74	0.09	0.56–0.93	0.02
	VAL	-	0.02	0.040	0.74	0.09	0.56–0.92	0.02
	TAU	-	0.01	0.040	0.77	0.08	0.59–0.94	0.01
	SPER	-	0.02	0.040	0.75	0.08	0.57–0.92	0.02

+ or - indicates a higher or lower level of the metabolite in RR vs PP group.

## Supplementary Materials

The following are available online at https://www.mdpi.com/2077-0383/9/3/863/s1, Figure S1: Multivariate analysis of CSF samples and serum samples with NMR and GC-MS. Figure S2: Graphs of the Spearman Correlation of the metabolites of CSF passing the Holm–Bonferroni correction. Figure S3: Graphs of the Spearman Correlation of the metabolites of serum passing the Holm–Bonferroni correction. Table S1: Summary of the analyte abbreviations of Biocrates. Table S2: Statistical parameters of the multivariate models resulting from the analysis of the matrix generated by NMR and GC-MS analysis.
