# Supplementary material for: Multi-Platform Characterization of Cerebrospinal Fluid and Serum Metabolome of Patients Affected by Relapsing–Remitting and Primary Progressive Multiple Sclerosis
Source: J Clin Med. 2020 Mar 21;9(3):863. doi: 10.3390/jcm9030863 (PMC7141510; doi:10.3390/jcm9030863)
Supplement: Supplementary file 1 [file jcm-09-00863-s001.pdf]

Table S1. Summary of the analyte abbreviations of Biocrates.

**Amino acids**	
**BC code**	**Analyte**
Ala	Alanine
Arg	Arginine
Asn	Asparagine
Asp	Aspartate
Cit	Citrulline
Gln	Glutamine
Glu	Glutamate
Gly	Glycine
His	Histidine
Ile	Isoleucine
Leu	Leucine
Lys	Lysine
Met	Methionine
Orn	Ornithine
Phe	Phenylalanine
Pro	Proline
Ser	Serine
Thr	Threonine
Trp	Tryptophan
Tyr	Tyrosine
Val	Valine
**Biogenic Amines**	
Ac-Orn	Acetylornithine
ADMA	Asymmetric dimethylarginine
SDMA	Symmetric dimethylarginine
alpha-AAA	alpha-Aminoadipic acid
Histamine	Histamine
Met-SO	Methionine-Sulfoxide
Kyn	Kynurenine
Putrescine	Putrescine
Spermidine	Spermidine
Spermine	Spermine
Serotonin	Serotonin
PEA	Phenylethylamine
Nitro-Tyr	Nitrotyrosine
c4-OH-Pro	cis-4-Hydroxyproline
t4-OH-Pro	trans-4-Hydroxyproline
Creatinine	Creatinine
Carnosine	Carnosine
Taurine	Taurine

DOPA	Dihydroxyphenylalanine
Dopamin	Dopamin
**Acylcarnitine**	
C0	Carnitine (free)
C2	Acetylcarnitine
C3	Propionylcarnitine
C3:1	Propenoylcarnitine
C3-OH	Hydroxypropionylcarnitine
C4	Butyrylcarnitine/Isobutyrylcarnitine
C4:1	Butenoylcarnitine
C4-OH (C3-DC)	Hydroxybutyrylcarnitine (Malonylcarnitine)
C5	Isovalerylcarnitine/2-Methylbutyrylcarnitine/Valerylcarnitine
C5:1	Tiglylcarnitine/3-Methyl-crotonylcarnitine
C5:1-DC	Glutaconylcarnitine/Mesaconylcarnitine
C5-DC (C6-OH)	Glutarylcarnitine (Hydroxyhexanoylcarnitine (= Hydroxycaproylcarnitine))
C5-M-DC	Methylglutarylcarnitine
C5-OH (C3-DC-M)	Hydroxyisovalerylcarnitine/Hydroxy-2-methylbutyryl/Hydroxyvalerylcarnitine (Methylmalonylcarnitine)
C6 (C4:1-DC)	Hexanoylcarnitine (= Caproylcarnitine) (Fumarylcarnitine)
C6:1	Hexenoylcarnitine
C7-DC	Pimelylcarnitine
C8	Octanoylcarnitine (= Caprylylcarnitine)
C9	Nonanoylcarnitine (= Pelargonylcarnitine)
C10	Decanoylcarnitine (= Caprylcarnitine)
C10:1	Decenoylcarnitine
C10:2	Decadienoylcarnitine
C12	Dodecanoylcarnitine (= Laurylcarnitine)
C12:1	Dodecenoylcarnitine
C12-DC	Dodecanedioylcarnitine
C14	Tetradecanoylcarnitine (= Myristylcarnitine)
C14:1	Tetradecenoylcarnitine (= Myristoleylcarnitine)
C14:1-OH	Hydroxytetradecenoylcarnitine (= Hydroxymyristoleylcarnitine)
C14:2	Tetradecadienoylcarnitine
C14:2-OH	Hydroxytetradecadienoylcarnitine
C16	Hexadecanoylcarnitine (= Palmitoylcarnitine)
C16:1	Hexadecenoylcarnitine (= Palmitoleylcarnitine)
C16:1-OH	Hydroxyhexadecenoylcarnitine (= Hydroxypalmitoleylcarnitine)
C16:2	Hexadecadienoylcarnitine
C16:2-OH	Hydroxyhexadecadienoylcarnitine
C16-OH	Hydroxyhexadecanolycarnitine (= Hydroxypalmitoylcarnitine)
C18	Octadecanoylcarnitine (= Stearylcarnitine)
C18:1	Octadecenoylcarnitine (= Oleylcarnitine)
C18:1-OH	Hydroxyoctadecenoylcarnitine (= Hydroxyoleylcarnitine)

C18:2	Octadecadienoylcarnitine (= Linoleylcarnitine)
**Lysophosphatidylcholines**	
lysoPC a C14:0	Lysophosphatidylcholine with acyl residue C14:0
lysoPC a C16:0	Lysophosphatidylcholine with acyl residue C16:0
lysoPC a C16:1	Lysophosphatidylcholine with acyl residue C16:1
lysoPC a C17:0	Lysophosphatidylcholine with acyl residue C17:0
lysoPC a C18:0	Lysophosphatidylcholine with acyl residue C18:0
lysoPC a C18:1	Lysophosphatidylcholine with acyl residue C18:1
lysoPC a C18:2	Lysophosphatidylcholine with acyl residue C18:2
lysoPC a C20:3	Lysophosphatidylcholine with acyl residue C20:3
lysoPC a C20:4	Lysophosphatidylcholine with acyl residue C20:4
lysoPC a C24:0	Lysophosphatidylcholine with acyl residue C24:0
lysoPC a C26:0	Lysophosphatidylcholine with acyl residue C26:0
lysoPC a C26:1	Lysophosphatidylcholine with acyl residue C26:1
lysoPC a C28:0	Lysophosphatidylcholine with acyl residue C28:0
lysoPC a C28:1	Lysophosphatidylcholine with acyl residue C28:1
**Phosphatidylcholines**	
PC aa C24:0	Phosphatidylcholine with diacyl residue sum C24:0
PC aa C26:0	Phosphatidylcholine with diacyl residue sum C26:0
PC aa C28:1	Phosphatidylcholine with diacyl residue sum C28:1
PC aa C30:0	Phosphatidylcholine with diacyl residue sum C30:0
PC aa C30:2	Phosphatidylcholine with diacyl residue sum C30:2
PC aa C32:0	Phosphatidylcholine with diacyl residue sum C32:0
PC aa C32:1	Phosphatidylcholine with diacyl residue sum C32:1
PC aa C32:2	Phosphatidylcholine with diacyl residue sum C32:2
PC aa C32:3	Phosphatidylcholine with diacyl residue sum C32:3
PC aa C34:1	Phosphatidylcholine with diacyl residue sum C34:1
PC aa C34:2	Phosphatidylcholine with diacyl residue sum C34:2
PC aa C34:3	Phosphatidylcholine with diacyl residue sum C34:3
PC aa C34:4	Phosphatidylcholine with diacyl residue sum C34:4
PC aa C36:0	Phosphatidylcholine with diacyl residue sum C36:0
PC aa C36:1	Phosphatidylcholine with diacyl residue sum C36:1
PC aa C36:2	Phosphatidylcholine with diacyl residue sum C36:2
PC aa C36:3	Phosphatidylcholine with diacyl residue sum C36:3
PC aa C36:4	Phosphatidylcholine with diacyl residue sum C36:4
PC aa C36:5	Phosphatidylcholine with diacyl residue sum C36:5
PC aa C36:6	Phosphatidylcholine with diacyl residue sum C36:6
PC aa C38:0	Phosphatidylcholine with diacyl residue sum C38:0
PC aa C38:1	Phosphatidylcholine with diacyl residue sum C38:1
PC aa C38:3	Phosphatidylcholine with diacyl residue sum C38:3
PC aa C38:4	Phosphatidylcholine with diacyl residue sum C38:4
PC aa C38:5	Phosphatidylcholine with diacyl residue sum C38:5
PC aa C38:6	Phosphatidylcholine with diacyl residue sum C38:6

PC aa C40:1	Phosphatidylcholine with diacyl residue sum C40:1
PC aa C40:2	Phosphatidylcholine with diacyl residue sum C40:2
PC aa C40:3	Phosphatidylcholine with diacyl residue sum C40:3
PC aa C40:4	Phosphatidylcholine with diacyl residue sum C40:4
PC aa C40:5	Phosphatidylcholine with diacyl residue sum C40:5
PC aa C40:6	Phosphatidylcholine with diacyl residue sum C40:6
PC aa C42:0	Phosphatidylcholine with diacyl residue sum C42:0
PC aa C42:1	Phosphatidylcholine with diacyl residue sum C42:1
PC aa C42:2	Phosphatidylcholine with diacyl residue sum C42:2
PC aa C42:4	Phosphatidylcholine with diacyl residue sum C42:4
PC aa C42:5	Phosphatidylcholine with diacyl residue sum C42:5
PC aa C42:6	Phosphatidylcholine with diacyl residue sum C42:6
PC ae C30:0	Phosphatidylcholine with acyl-alkyl residue sum C30:0
PC ae C30:1	Phosphatidylcholine with acyl-alkyl residue sum C30:1
PC ae C30:2	Phosphatidylcholine with acyl-alkyl residue sum C30:2
PC ae C32:1	Phosphatidylcholine with acyl-alkyl residue sum C32:1
PC ae C32:2	Phosphatidylcholine with acyl-alkyl residue sum C32:2
PC ae C34:0	Phosphatidylcholine with acyl-alkyl residue sum C34:0
PC ae C34:1	Phosphatidylcholine with acyl-alkyl residue sum C34:1
PC ae C34:2	Phosphatidylcholine with acyl-alkyl residue sum C34:2
PC ae C34:3	Phosphatidylcholine with acyl-alkyl residue sum C34:3
PC ae C36:0	Phosphatidylcholine with acyl-alkyl residue sum C36:0
PC ae C36:1	Phosphatidylcholine with acyl-alkyl residue sum C36:1
PC ae C36:2	Phosphatidylcholine with acyl-alkyl residue sum C36:2
PC ae C36:3	Phosphatidylcholine with acyl-alkyl residue sum C36:3
PC ae C36:4	Phosphatidylcholine with acyl-alkyl residue sum C36:4
PC ae C36:5	Phosphatidylcholine with acyl-alkyl residue sum C36:5
PC ae C38:0	Phosphatidylcholine with acyl-alkyl residue sum C38:0
PC ae C38:1	Phosphatidylcholine with acyl-alkyl residue sum C38:1
PC ae C38:2	Phosphatidylcholine with acyl-alkyl residue sum C38:2
PC ae C38:3	Phosphatidylcholine with acyl-alkyl residue sum C38:3
PC ae C38:4	Phosphatidylcholine with acyl-alkyl residue sum C38:4
PC ae C38:5	Phosphatidylcholine with acyl-alkyl residue sum C38:5
PC ae C38:6	Phosphatidylcholine with acyl-alkyl residue sum C38:6
PC ae C40:1	Phosphatidylcholine with acyl-alkyl residue sum C40:1
PC ae C40:2	Phosphatidylcholine with acyl-alkyl residue sum C40:2
PC ae C40:3	Phosphatidylcholine with acyl-alkyl residue sum C40:3
PC ae C40:4	Phosphatidylcholine with acyl-alkyl residue sum C40:4
PC ae C40:5	Phosphatidylcholine with acyl-alkyl residue sum C40:5
PC ae C40:6	Phosphatidylcholine with acyl-alkyl residue sum C40:6
PC ae C42:0	Phosphatidylcholine with acyl-alkyl residue sum C42:0
PC ae C42:1	Phosphatidylcholine with acyl-alkyl residue sum C42:1
PC ae C42:2	Phosphatidylcholine with acyl-alkyl residue sum C42:2

PC ae C42:3	Phosphatidylcholine with acyl-alkyl residue sum C42:3
PC ae C42:4	Phosphatidylcholine with acyl-alkyl residue sum C42:4
PC ae C42:5	Phosphatidylcholine with acyl-alkyl residue sum C42:5
PC ae C44:3	Phosphatidylcholine with acyl-alkyl residue sum C44:3
PC ae C44:4	Phosphatidylcholine with acyl-alkyl residue sum C44:4
PC ae C44:5	Phosphatidylcholine with acyl-alkyl residue sum C44:5
PC ae C44:6	Phosphatidylcholine with acyl-alkyl residue sum C44:6
**Sphingomyelins Analysis**	
SM (OH) C14:1	Hydroxysphingomyelin with acyl residue sum C14:1
SM (OH) C16:1	Hydroxysphingomyelin with acyl residue sum C16:1
SM (OH) C22:1	Hydroxysphingomyelin with acyl residue sum C22:1
SM (OH) C22:2	Hydroxysphingomyelin with acyl residue sum C22:2
SM (OH) C24:1	Hydroxysphingomyelin with acyl residue sum C24:1
SM C16:0	Sphingomyelin with acyl residue sum C16:0
SM C16:1	Sphingomyelin with acyl residue sum C16:1
SM C18:0	Sphingomyelin with acyl residue sum C18:0
SM C18:1	Sphingomyelin with acyl residue sum C18:1
SM C20:2	Sphingomyelin with acyl residue sum C20:2
SM C22:3	Sphingomyelin with acyl residue sum C22:3
SM C24:0	Sphingomyelin with acyl residue sum C24:0
SM C24:1	Sphingomyelin with acyl residue sum C24:1
SM C26:0	Sphingomyelin with acyl residue sum C26:0
SM C26:1	Sphingomyelin with acyl residue sum C26:1
**Hexoses**	
H1	Glucose
H1	Aldohexose
H1	L-Allopyranose
H1	D-Allose
H1	D-Allopyranose
H1	D-Allose
H1	D-Altropyranose
H1	D-Glucopyranose
H1	alpha-D-Glucopyranose
H1	beta-D-Glucopyranose
H1	D-Mannopyranose
H1	alpha-D-Mannopyranose
H1	L-Gulopyranose
H1	D-Gulopyranose
H1	D-Idopyranose
H1	Alpha-L-Galactopyranose
H1	alpha-D-Galactopyranose
H1	beta-D-Galactopyranose
H1	D-Talose

H1	D-Talopyranose
H1	Ketohexose
H1	D-Psicopyranose
H1	L-Fructofuranose
H1	D-Fructose
H1	D-Fructofuranose
H1	L-Sorbopyranose
H1	D-Sorbopyranose
H1	D-Tagatose
H1	D-Tagatopyranose

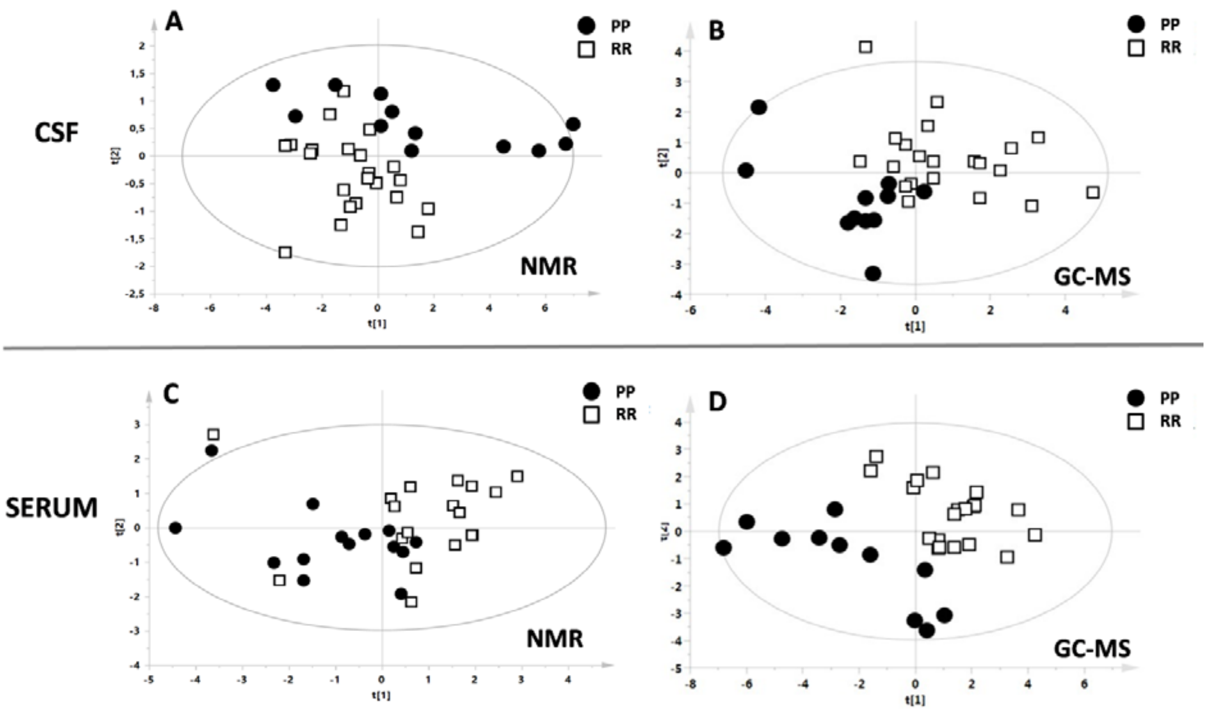

Figure S1. Multivariate analysis. **A–B**: models resulted from the analysis of cerebrospinal fluid (CSF) samples with NMR and GC-MS. The statistical parameters were not significant. **C–D**: models resulted from the analysis of serum samples with NMR and GC-MS. Black circles indicate PPMS patients while white boxes indicate RRMS patientsThe statistical parameters were not significant.

Table S2. Statistical parameters of the multivariate models resulting from the analysis of the matrix generated by NMR and GC-MS analysis

	CSF		SERUM	
	Q^2	*p*-value	Q^2	*p*-value
**NMR**	0.01	0.8	-0.001	1
**GC-MS**	0.12	0.21	0.223	0.13

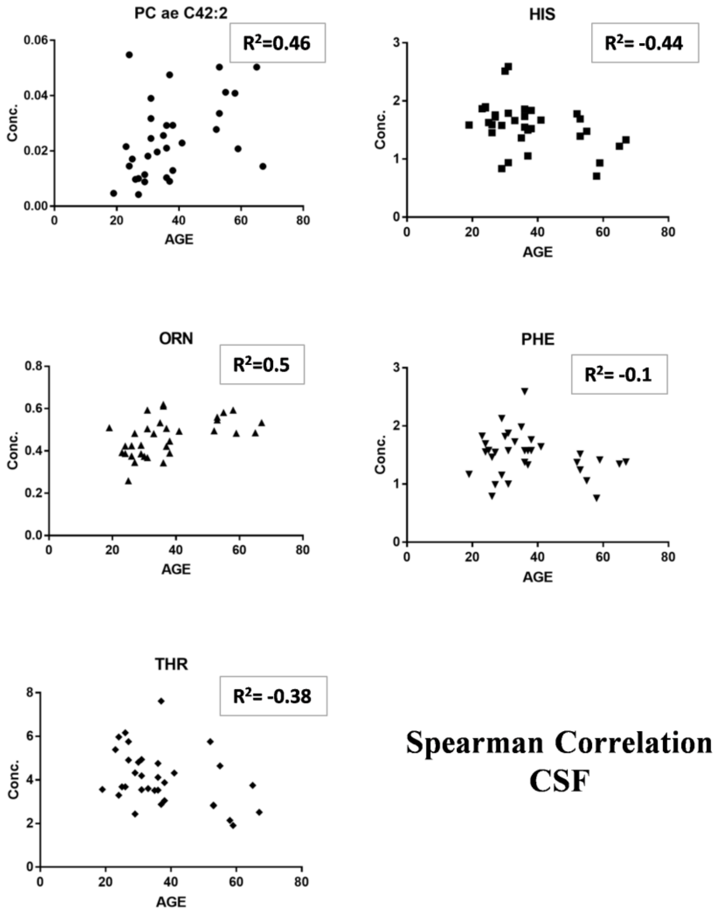

Figure S2. Graphs of the Spearman Correlation of the metabolites of CSF passing the Holm–Bonferroni correction. R^2 is reportedfor each graph.

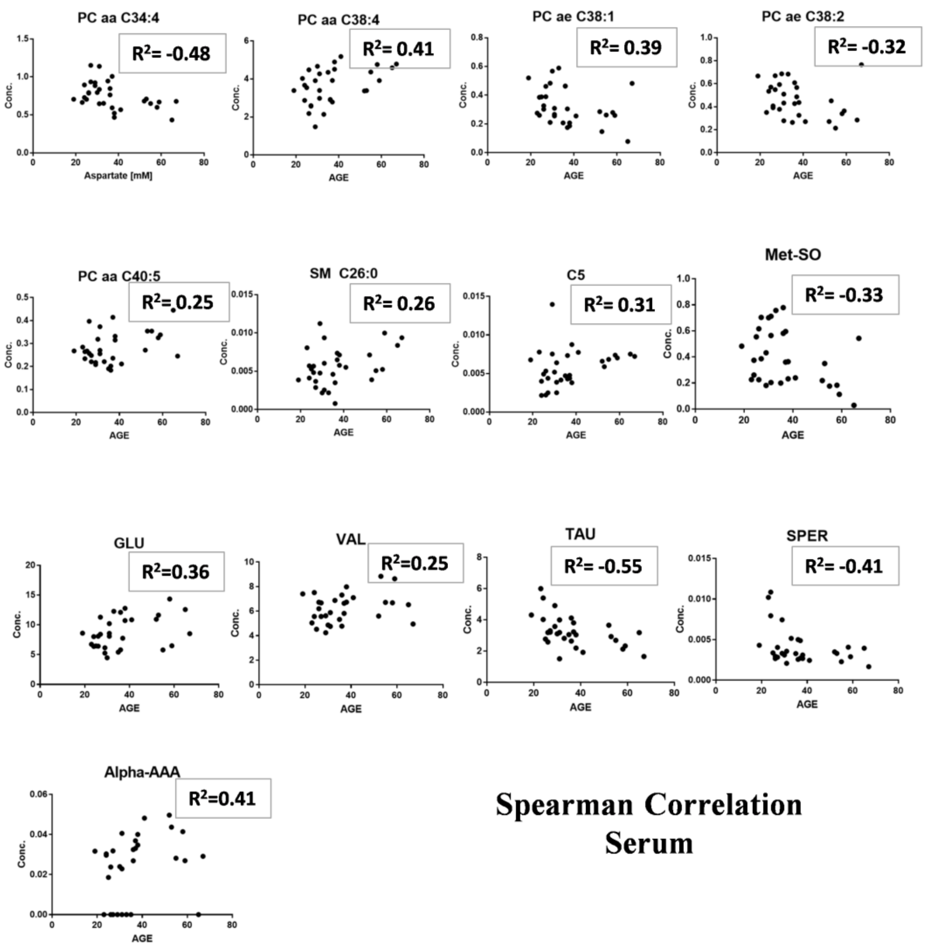

Figure S3. Graphs of the Spearman Correlation of the metabolites of serum passing the Holm–Bonferroni correction. R^2 is reported for each graph.
